# Predictors of ventilator-associated pneumonia in intubated pediatric trauma patients

**DOI:** 10.1007/s00383-025-06131-6

**Published:** 2025-07-17

**Authors:** Fnu Avinash, Jeffry Nahmias, Negaar Aryan, James Jeng, Cristobal Barrios, Peter D. Nguyen, Areg Grigorian

**Affiliations:** Department of Surgery, Division of Division of Trauma, Burns and Surgical Critical Care, University of California, Irvine Medical Center, 3800 Chapman Ave, Suite 6200, Orange, Irvine, CA 92868-3298 USA

**Keywords:** Ventilator-associated pneumonia, VAP, Pediatric trauma, Pediatric intensive care, Pulmonary complication

## Abstract

**Purpose:**

Ventilator-associated pneumonia (VAP) is the most common complication among intubated pediatric trauma patients (PTPs) in pediatric intensive care units. Early identification of associated risk factors may help mitigate adverse outcomes linked to VAP, such as increased mortality and healthcare costs. This study aims to identify risk factors associated with VAP for intubated PTPs.

**Methods:**

The 2017–2021 Trauma Quality Improvement Program database was queried for all intubated PTPs. Two groups were compared: intubated PTPs with and without VAP. Bivariate and multivariable logistic regression analyses were performed.

**Results:**

From 38,593 intubated PTPs, 819 (2.1%) developed VAP. The VAP cohort had a higher injury severity score with increased rates of traumatic brain injury (TBI) (75.3% vs. 55.4%, *p* < 0.001), rib fractures (24.0% vs. 16.4%, *p* < 0.001), and lung injuries (20.8% vs. 10.6%, *p* < 0.001). Independent associated risk factors for VAP included unplanned reintubation (OR 2.51, CI 1.84–3.43, *p* < 0.001), TBI (OR 1.96, CI 1.63–2.36, *p* < 0.001), and severe thoracic injury (OR 1.27, CI 1.01–1.58, *p* < 0.001).

**Conclusion:**

Unplanned reintubation, TBI, and severe thoracic injuries are key risk factors for VAP in intubated PTPs. Our findings highlight the need for strategies to reduce reintubation, optimize ventilator management, and improve pulmonary care in high-risk PTPs.

**Level of evidence:**

IV.

**Supplementary Information:**

The online version contains supplementary material available at 10.1007/s00383-025-06131-6.

## Introduction

Trauma remains a leading cause of morbidity and mortality in pediatric populations, sometimes necessitating intensive care management [1]. Among critically ill children, infections significantly contribute to poor outcomes and increased healthcare costs, with up to 12% of patients admitted to pediatric intensive care units (PICUs) developing one or more infections during their admission [2]. Mechanical ventilation is routinely utilized in PICUs, with over 20% of patients requiring invasive ventilator support [3]. However, prolonged mechanical ventilation poses risks, including ventilator-associated pneumonia (VAP) [1, 3]. The rate of VAP in pediatric trauma patients (PTPs) varies from 1 to 10% but can be as high as 40% in patients with traumatic brain injury (TBI) [4]. VAP is associated with prolonged mechanical ventilation, which increases total hospital length of stay (LOS), mortality, and healthcare costs [5].

While the incidence of VAP in children is substantially lower than in adults, it remains a major source of secondary morbidity in PTPs [1]. Known risk factors for VAP in general PICU populations include pre-existing medical conditions (cerebral palsy, cystic fibrosis, and immunosuppression), prolonged mechanical ventilation, and chronic hospitalizations [6–8]. However, identifying risk factors in PTPs is important for risk stratification and management. This study aims to analyze the incidence of VAP in intubated PTPs and identify associated risk factors.

## Methods

This study was approved by our Institutional Review Board and a waiver of consent was granted as it utilizes a national deidentified database. The 2017–2021 Trauma Quality Improvement Program (TQIP) database was queried for PTPs 17 years of age and younger who underwent intubation. This was defined by any patient that had a ventilator day recorded in the TQIP database [6].

The primary outcome was the incidence of VAP. TQIP defines VAP in a patient on mechanical ventilation for > 2 days with radiological evidence of new or progressive infiltrate, symptomatic evidence of systemic infection, and laboratory detection of a causative agent [6]. We compared two groups: PTPs who developed VAP and those without VAP. Demographic variables collected included age, sex, vitals on arrival, and comorbidities such as mental illness, diabetes mellitus, hypertension, smoking status, and substance use disorder. Mental illness is defined as a pre-existing diagnosis of any personality disorder, obsessive–compulsive disorder, or panic disorder. The injury profile included the injury severity score (ISS) and specific injuries to the brain, spinal cord, rib fracture, lungs, liver, spleen, stomach, intestines, kidney, pelvis, as well as the upper and lower extremities. Severe thoracic injury was defined by an abbreviated injury scale grade > 3 for the thorax. Secondary outcomes included the total hospital LOS, ICU LOS, ventilator days, and the mortality rate. Additionally, inpatient complications were analyzed, including cerebrovascular accident (CVA), acute respiratory distress syndrome (ARDS), pulmonary embolism (PE), cardiac arrest, acute kidney injury, surgical site infections, deep vein thrombosis (DVT), pressure ulcer, catheter-associated urinary tract infection, central line-associated bloodstream infection, unplanned reintubation, and unplanned return to OR.

Bivariate analyses were performed using a Mann–Whitney U test to compare continuous variables and Pearson’s chi-squared analysis to compare categorical variables. Categorical data were reported as percentages and continuous data were reported as medians with interquartile range or as means with standard deviation. A multivariable logistic regression model was used to determine the risk of VAP in PTPs. After a discussion among coauthors and a review of the literature, we selected variables available in TQIP that are associated with the risk of VAP to include in the model: age, hypotension on admission (systolic blood pressure < 90 mmHg), ISS, blood transfusion, unplanned reintubation, emergent operation, TBI, severe thoracic injury, and steroid use [1, 4, 7, 9, 10]. Additionally, a sensitivity analysis was conducted using a multivariable model that included only pre-intubation covariates: age, hypotension on admission (systolic blood pressure < 90 mmHg), ISS, TBI, severe thoracic injury, and home steroid use. The multivariable logistic regression model reported the risk of VAP with an odds ratio (OR) and 95% confidence intervals (CI). All *p *values were two-sided with a statistical significance level of < 0.05. All analyses were conducted using IBM SPSS Statistics for Windows (version 29; IBM Corporation, Armonk, NY).

## Results

### Demographics, and comorbidities of intubated pediatric patients with and without VAP

From 38,593 intubated PTPs, 819 (2.1%) developed VAP. The median age was 16 in the VAP group versus 13 in the non-VAP group (*p* < 0.001). Compared to patients without VAP, PTPs with VAP had higher rates of smoking (3.8% vs. 1.9%, *p* < 0.001) and substance use disorder (4.9% vs. 2.4%, *p* < 0.001). The VAP group had a lower rate of tachypnea upon arrival (39.7% vs. 46.7%, %, *p* < 0.001), compared to the non-VAP group (Table 1).

**Table 1:** Demographics for intubated pediatric patients with and without VAP

Characteristic	VAP ( +)	VAP (–)	*p *value
	(*n* = 819)	(*n* = 37,774)	
Age, year, median (IQR)	16 (13, 17)	13 (6, 16)	** < 0.001**
Male, *n* (%)	577 (70.5%)	25,587 (68.0%)	0.118
Comorbidities, *n* (%)			
Mental illness^a^	38 (4.8%)	1,520 (4.1%)	0.359
Hypertension	5 (0.6%)	144 (0.4%)	0.289
Diabetes	6 (0.8%)	143 (0.4%)	0.103
Smoking	30 (3.8%)	712 (1.9%)	** < 0.001**
Substance use disorder	39 (4.9%)	882 (2.4%)	** < 0.001**
Vitals on arrival, *n* (%)			
Hypotensive (SBP < 90 mmHg)	112 (14.0%)	5,608 (15.4%)	0.269
Tachycardia (HR > 120/min)	296 (36.7%)	14,166 (38.3%)	0.364
Tachypnea (RR > 22/min)	306 (39.7%)	16,541 (46.7%)	** < 0.001**

*VAP: ventilator-associated pneumonia, IQR: interquartile range, SBP: systolic blood pressure, HR: heart rate, RR: respiratory rate*

^*a*^*Defined as any personality, obsessive–compulsive, or panic disorder*

### Injury profile of intubated pediatric patients with and without VAP

PTPs with VAP had a higher median ISS (29 vs. 21, *p* < 0.001) compared to the non-VAP group. PTPs who developed VAP had higher rates of TBI (75.3% vs. 55.4%, *p* < 0.001), spinal fracture (36.0% vs. 19.2%, *p* < 0.001), lung injuries (56.0% vs. 37.2%, *p* < 0.001), and rib fractures (25.2% vs. 15.0%, *p* < 0.001), compared to PTPs without VAP (Table 2).

**Table 2:** Injuries of intubated pediatric patients with and without VAP

Characteristic	VAP ( +)	VAP (–)	*p *value
	(*n* = 819)	(*n* = 37,774)	
ISS, median (IQR)	29 (24, 38)	21 (11, 29)	** < 0.001**
Injury, *n* (%)			
TBI	617 (75.3%)	20,940 (55.4%)	** < 0.001**
Cervical cord	72 (8.8%)	1,145 (3.0%)	** < 0.001**
Spine fracture	295 (36.0%)	7,252 (19.2%)	** < 0.001**
Rib fracture	206 (25.2%)	5,673 (15.0%)	** < 0.001**
Lung	459 (56.0%)	14,049 (37.2%)	** < 0.001**
Cardiac	22 (2.7%)	769 (2.0%)	0.194
Diaphragm	24 (2.9%)	889 (2.4%)	0.282
Liver	128 (15.6%)	4,657 (12.3%)	**0.005**
Spleen	122 (14.9%)	3,516 (9.3%)	** < 0.001**
Esophagus	0 (0%)	86 (0.2%)	0.172
Stomach	9 (1.1%)	632 (1.7%)	0.203
Kidney	74 (9.0%)	2,349 (6.2%)	** < 0.001**
Bladder	11 (1.3%)	350 (0.9%)	0.221
Small intestine	29 (3.5%)	1,882 (5.0%)	0.060
Colon	35 (4.3%)	1,617 (4.3%)	0.992
Rectum	2 (0.2%)	189 (0.5%)	0.301
Pelvis	151 (18.4%)	4022 (10.6%)	** < 0.001**

*VAP: ventilator-associated pneumonia, ISS: injury severity score, IQR: interquartile range,* TBI*: traumatic brain injury*

### Clinical outcomes of intubated pediatric patients with and without VAP

PTPs in the VAP cohort had increased median ICU LOS (20 vs. 4 days, *p* < 0.001) and ventilator days (15 vs. 2 days, *p* < 0.001), compared to PTPs without VAP. Additionally, PTPs with VAP also had higher rates of hospital complications including ARDS (11.6% vs. 1.1%, *p* < 0.001), PE (3.1% vs. 0.3%, *p* < 0.001), pressure ulcers (15.1% vs. 2.0%, *p* < 0.001), unplanned reintubation (6.9% vs. 1.9%, *p* < 0.001), and unplanned return to OR (8.3% vs. 2.6%, *p* < 0.001). However, PTPs with VAP had a lower mortality rate than PTPs without VAP (8.7% vs. 18.1%, *p* < 0.001) (Table 3).

**Table 3:** Clinical outcomes of intubated pediatric patients who developed VAP vs. those who never developed VAP

Characteristic	VAP ( +)	VAP (–)	*p*-value
	(*n* = 819)	(*n* = 37,774)	
ICU LOS, days, median (IQR)	20 (14, 30)	4 (2, 9)	** < 0.001**
Ventilator, days, median (IQR)	15 (10, 24)	2 (1,5)	** < 0.001**
Complications, *n* (%)			
Cerebrovascular accident	24 (3.0%)	231 (0.6%)	** < 0.001**
ARDS	94 (11.6%)	400 (1.1%)	** < 0.001**
Pulmonary embolism	25 (3.1%)	129 (0.3%)	** < 0.001**
Cardiac arrest	49 (6.0%)	1,485 (3.9%)	**0.002**
Acute kidney injury	36 (4.4%)	319 (0.8%)	** < 0.001**
Sepsis	42 (5.2%)	146 (0.4%)	** < 0.001**
Superficial incision SSI	17 (2.1%)	142 (0.4%)	** < 0.001**
Deep SSI	16 (2.0%)	151 (0.4%)	** < 0.001**
Organ space SSI	12 (1.5%)	139 (0.4%)	** < 0.001**
Deep vein thrombosis	76 (9.3%)	553 (1.5%)	** < 0.001**
Pressure ulcer	123 (15.1%)	763 (2.0%)	** < 0.001**
CAUTI	50 (6.2%)	195 (0.5%)	** < 0.001**
CLABSI	10 (1.2%)	66 (0.2%)	** < 0.001**
Unplanned reintubation	56 (6.9%)	713 (1.9%)	** < 0.001**
Unplanned trip to OR	67 (8.3%)	944 (2.6%)	** < 0.001**
Emergent OR	242 (29.5%)	9,621 (25.5%)	**0.008**
Mortality, n (%)	71 (8.7%)	6,824 (18.1%)	** < 0.001**

*VAP: ventilator-associated pneumonia, ICU: intensive care unit, LOS: length of stay, IQR: interquartile range, ARDS: acute respiratory distress syndrome, SSI: surgical site infection, CAUTI: catheter-associated urinary tract infection, CLABSI: central line-associated blood stream infection, OR: operating room*

On multivariable logistic regression analysis, after adjusting for known predictors, the strongest independent associated risk factor for VAP was unplanned reintubation (OR 2.51, CI 1.84–3.43, *p* < 0.001), TBI (OR 1.96, CI 1.63–2.36, *p* < 0.01), and severe thoracic injury (OR 1.27, CI 1.01–1.58, *p* = 0.03) (Table 4). On sensitivity analysis, TBI (OR 1.85, CI 1.56–2.20, *p* < 0.001) remained the strongest independent associated risk factor for the development of VAP (Supplementary Table 1).

**Table 4:** Multivariable logistical regression analysis for risk of developing VAP

Predictors of VAP	OR	CI	*p*-value
Age, years	1.13	1.10–1.15	** < 0.001**
Hypotension on arrival (SBP < 90 mmHg)	0.82	0.65–1.03	0.092
Injury severity score	1.02	1.01–1.02	** < 0.001**
PRBC transfusion	0.89	0.74–1.08	0.257
Total ventilator days	1.05	1.05–1.06	** < 0.001**
Unplanned reintubation	2.51	1.84–3.43	** < 0.001**
Emergent operation	1.07	0.90–1.28	0.397
Steroid use	1.13	0.15–8.44	0.900
Injuries			
Traumatic brain injury	1.96	1.63–2.36	** < 0.001**
Severe thoracic injury	1.27	1.01–1.58	**0.037**

*VAP: ventilator-associated pneumonia, SBP: systolic blood pressure, PRBC: packed red blood cells*

## Discussion

Our study identified key predictors of VAP in intubated PTPs, including unplanned reintubation, TBI, and severe thoracic injury. Additionally, our results demonstrated that patients with VAP experienced markedly longer durations of mechanical ventilation and ICU stays, as well as a higher incidence of complications such as ARDS, DVT, and pressure ulcers compared to those without VAP.

In adult and PTPs, the need for reintubation often arises due to airway obstruction, poor respiratory mechanics, or deteriorating clinical status [10, 11]. Unplanned reintubation has been consistently identified as a significant risk factor for the development of VAP in both adult and pediatric populations [5, 11, 12]. In support of this, we also identified unplanned reintubation to be associated with a higher risk of VAP. The link between reintubation and VAP can be attributed to several mechanisms [11, 12]. Each intubation event disrupts the airway mucosa, increasing vulnerability to bacterial colonization [13]. The process of reintubation also increases the risk of aspiration due to compromised airway protection, leading to the introduction of oropharyngeal and gastric contents into the lower respiratory tract [13, 19]. Prior studies have demonstrated that reintubation prolongs the duration of mechanical ventilation, which in turn extends the exposure to ventilator-associated risks such as impaired mucociliary clearance and biofilm formation on the endotracheal tube [14, 16]. Our study confirmed this association, as PTPs with VAP had significantly longer ventilator days compared to those without VAP.

Increased ventilator days and unplanned reintubation likely have a bidirectional relationship, where each factor amplifies the risk of the other [17]. Prolonged mechanical ventilation increases the likelihood of VAP by providing an extended opportunity for bacterial colonization, impairing mucociliary clearance, and promoting ventilator-associated lung injury [13]. Conversely, once VAP develops, patients often require longer ventilator support due to impaired respiratory function, which in turn increases the risk of complications, such as atelectasis and barotrauma [17]. In our study, it is unclear whether prolonged ventilation was a driving factor for VAP, or whether VAP itself led to extended ventilator days. This extended ICU course and ventilator dependence may also explain the higher rate of pressure ulcers observed in the VAP cohort. Pressure ulcers are associated with prolonged immobilization and sedation, both of which are common in patients with VAP [17]. This extended ICU course and ventilator dependence may also explain the significantly higher rate of pressure ulcers observed in the VAP cohort. PTPs with VAP were over seven times more likely to develop pressure ulcers compared to those without VAP, reflecting the broader systemic burden of prolonged critical illness. This finding highlights the need for early mobilization, frequent repositioning, and preventive skin care to reduce secondary complications in high-risk PTPs.

Additionally, unplanned reintubation is often a marker of failed extubation, which may indicate underlying neuromuscular weakness, inadequate pulmonary reserve, or persistent respiratory distress [20]. When combined with prolonged mechanical ventilation, repeated intubation creates a compounding effect that increases the risk of VAP, establishing a vicious cycle of respiratory compromise [10]. These findings highlight the need to minimize reintubation events through careful extubation planning. This includes the use of objective weaning criteria, adjunctive strategies, such as noninvasive ventilation, and pulmonary hygiene measures [10]. Standard weaning parameters, such as the negative inspiratory force (NIF) and rapid shallow breathing index (RSBI), have not consistently been shown to be associated with successful extubation in PTPs, and other factors, such as age and ISS, may be more important [17]. Age is an important factor in pediatric patients, as both physiologic and anatomic characteristics vary significantly across developmental stages. Therefore, future research should aim to identify age-specific optimal weaning protocols for PTPs to mitigate the risk of VAP while minimizing the incidence of premature extubation failures.

TBI is another significant risk factor for the development of VAP in PTPs, particularly in those requiring prolonged mechanical ventilation in the PICU [4]. In our study, two-thirds of intubated PTPs who developed VAP had TBI, reinforcing prior findings that impaired airway protection, diminished cough reflex, and prolonged ventilator dependence contribute to infection risk. TBI can disrupt proper secretion clearance, predisposing patients to VAP [4]. Therefore, these risk factors act as confounders, since PTPs with TBI may experience silent aspiration, resulting in respiratory compromise that necessitates unplanned reintubation. Additionally, interventions such as sedation and neuromuscular blockade, often necessary for TBI management, further delay extubation and impair pulmonary function, compounding the risk [15, 18, 21]. The development of VAP in this cohort is likely multifactorial, driven by both the direct effects of brain injury on respiratory control and extended ICU stays [4, 22]. Given these risks, targeted prevention strategies are important. Early mobilization, aggressive pulmonary hygiene, and maintaining a semi-recumbent position have been shown to mitigate VAP risk and should be prioritized in PTPs with TBI [3, 23].

Severe thoracic injuries, including rib fractures and pulmonary contusions, are significant risk factors for VAP in PTPs, often occurring alongside TBI [24]. Unique pediatric anatomical features, such as increased chest wall compliance and smaller airway caliber, make children more vulnerable to respiratory complications following thoracic trauma [25]. Unlike adults, pediatric patients are more likely to sustain lung contusions without overt rib fractures, potentially leading to underestimated injury severity and delayed respiratory interventions [26, 27]. This increases the risk of atelectasis, aspiration, and pneumonia, all of which contribute to VAP [27]. Rib fractures and pulmonary contusions impair lung function by reducing chest wall movement, disrupting alveolar integrity, and promoting pulmonary edema, all of which prolong mechanical ventilation and increase the risk of bacterial colonization [20, 24, 28–30]. Additionally, thoracic trauma in PTPs is often part of multisystem injuries requiring extended immobilization and ventilatory support, further compounding VAP risk [31]. In our study, more than 50% of PTPs with VAP had lung injuries, aligning with findings by Brown et al., who reported a 1.5-fold increased VAP risk in pediatric patients with severe thoracic trauma [31].

Limitations to this study include those inherent to retrospective database studies, such as coding error, missing data, selection bias, and reporting bias. This study utilized the TQIP-defined criteria for VAP, which may differ from definitions used at non-TQIP participating institutions. However, all medical centers contributing data to TQIP adhere to standardized and published diagnostic guidelines, which supports the generalizability of these findings. Additionally, TQIP lacks detailed variables, such as ventilator settings, including tidal volumes, and peak pressures. This database study also lacks granular data pertaining to the timing of interventions, including the duration of intubation before reintubation or extubation attempts, thus limiting our ability to assess the temporal relationships between clinical events and the development of VAP. Also, the TQIP database only includes index hospitalization data and thus may underestimate mortality and complications. Despite certain limitations, the findings of this study are strengthened by a large patient population and multiple trauma centers, which enhances the generalizability of the findings in comparison to prior studies.

## Conclusions

Unplanned reintubation, TBI, and severe thoracic injuries are key risk factors for VAP in intubated PTPs. Our findings highlight the need for strategies to reduce reintubation, optimize ventilator management, and improve pulmonary care in high-risk PTPs. Targeted interventions, including structured weaning protocols, aggressive pulmonary hygiene, and early mobilization, may help mitigate VAP risk. Future prospective studies are necessary to confirm these associations and refine prevention strategies in pediatric trauma populations.

## Supplementary Information

Below is the link to the electronic supplementary material.

Supplementary file 1: Table 1. Multivariable logistical regression analysis for risk of developing VAP with pre-intubation covariates

## Data Availability

No datasets were generated or analyzed during the current study.
