# Supplementary material for: Predictors of ventilator-associated pneumonia in intubated pediatric trauma patients
Source: Pediatr Surg Int. 2025 Jul 17;41(1):216. doi: 10.1007/s00383-025-06131-6 (PMC12271297; doi:10.1007/s00383-025-06131-6)
Supplement: Supplementary file 1 — Table 1. Multivariable logistical regression analysis for risk of developing VAP with pre-intubation covariates [file 383_2025_6131_MOESM1_ESM.docx]

Supplementary Table 1. Multivariable logistical regression analysis for risk of developing VAP with pre-intubation covariates

| **Predictors of VAP** | **OR** | **CI** | **P-value** | |
| --- | --- | --- | --- | --- |
| Age, years | 1.11 | 1.09-1.13 | **<0.001** | |
| Hypotension on arrival (SBP <90mmHg) | 0.76 | 0.61-0.94 | **0.012** | |
| Injury severity score | 1.04 | 1.03-1.04 | **<0.001** | |
| Steroid use | 0.98 | 0.13-7.28 | 0.985 | |
| Traumatic brain injury | 1.85 | 1.56-2.20 | **<0.001** | |
| Severe thoracic injury | 1.06 | 0.86-1.31 | 0.587 | |
| *VAP = ventilator-associated pneumonia, SBP = systolic blood pressure* | | | |  |
